# In vivo impact of tubulin polymerization promoting protein (Tppp) knockout to the airway inflammatory response

**DOI:** 10.1038/s41598-023-39443-5

**Published:** 2023-07-28

**Authors:** Tori Endres, Lori Duesler, Deborah A. Corey, Thomas J. Kelley

**Affiliations:** 1 Department of Pediatrics, Case Western Reserve University, Cleveland, USA; 2 Department of Genetics and Genome Sciences, Case Western Reserve University, 825 BRB, 10900 Euclid Avenue, Cleveland, OH 44106-4955 USA; 3 Rainbow Babies and Children’s Hospital, Cleveland, OH USA

**Keywords:** Immunology, Physiology

## Abstract

Microtubule dysfunction has been implicated as a mediator of inflammation in multiple diseases such as disorders of the cardiovascular and neurologic systems. Tubulin polymerization promoting protein (Tppp) facilitates microtubule elongation and regulates tubulin acetylation through inhibition of cytosolic deacetylase enzymes. Pathologic alterations in microtubule structure and dynamics have been described in cystic fibrosis (CF) and associated with inflammation, however the causality and mechanism remain unclear. Likewise, Tppp has been identified as a potential modifier of CF airway disease severity. Here we directly assess the impact of microtubule dysfunction on infection and inflammation by interrogating wild type and a Tppp knockout mouse model (*Tppp − / −*). Mice are challenged with a clinical isolate of *Pseudomonas aeruginosa*-laden agarose beads and assessed for bacterial clearance and inflammatory markers. *Tppp − / − *mouse model demonstrate impaired bacterial clearance and an elevated inflammatory response compared to control mice. These data are consistent with the hypothesis microtubule dysregulation is sufficient to lead to CF-like airway responses in mice.

## Introduction

Microtubule stability is tightly regulated through post translational modifications including acetylation and deacetylation^1^. A hyper-inflammatory state has been described in a variety of genetic, cardiovascular, and neurologic conditions^2–4^. Specifically, microtubule dysfunction has been implicated as a mediator of the hyper-inflammatory response^5,6^. Cystic fibrosis (CF), an autosomal recessive genetic condition resulting from dysfunction of the cystic fibrosis transmembrane conductance regulator (CFTR), is associated with a hyperinflammatory state with elevated pro-inflammatory cytokines and neutrophilic predominant infiltrates^7^. Microtubule dynamics in CF have been well characterized and suggest a link between microtubule dysfunction and inflammation^8–10^. Structural defects in CF microtubules, including decreased acetylation and impairment of microtubule dynamic instability, result in functional impairment as evidenced by decreased intracellular endosomal trafficking^11^. Microtubule structural and functional deficits improve with restoration of microtubule acetylation, achieved through inhibition of a microtubule modifying protein, histone deacetylase 6 (HDAC6) or through stimulation of exchange protein activated by cAMP^5,12^. The inflammatory cytokine Mip-2, a cytokine produced by monocytes and epithelial cells, is reduced to WT levels in HDAC6 depleted mice. This observation is suggestive of a causal relationship between microtubule dysfunction and inflammation.

Functional outcomes of microtubule disruption have also been assessed in vivo. A mouse model of HDAC6 depletion shows attenuation of the negative sequelae of CFTR dysfunction following bacterial challenge with *Pseudomonas aeruginosa*^5^. Weight loss in response to bacterial challenge is lessened, bacterial levels are reduced at a faster rate, and percent neutrophils in bronchial alveolar lavage fluid are returned to WT levels in HDAC6 depleted mice. It has also been shown that anti-inflammatory therapies are able to correct microtubule stability in CF cells further linking the microtubule phenotype with inflammatory control. Ibuprofen has been shown to restore microtubule reformation rates, microtubule extension into the cell periphery, and restore intracellular trafficking as measured by cholesterol transport through and AMP-activated kinase-dependent mechanism^9^. Similarly, resveratrol, a known anti-inflammatory molecule, was shown to restore microtubule dynamics and intracellular trafficking partially through pan inhibition of histone deacetylases^13^.

Previous data demonstrate clearly that HDAC6 inhibition corrects inflammatory signaling in immortalized epithelial cell models of CF and in CF mouse models^5,8,14–16^. Though microtubule dysfunction in CF is corrected by HDAC6 inhibition, it is not clear that microtubule function is responsible for inflammation in CF since HDAC6 does have other targets^17–19^. Tubulin polymerization-promoting protein (Tppp) is a regulator of microtubule polymerization and promotes acetylation of alpha tubulin by binding to and inhibiting HDAC6^20^. We have previously shown that knocking down expression of Tppp in a human tracheal epithelial cell line increases inflammatory signaling consistent with what is seen in CF cells^12^. However, it is unclear how an in vivo depletion of Tppp expression will impact airway inflammatory responses to infection. Observing the phenotype of a mouse lacking expression of Tppp (*Tppp − / − *model) investigates the role of microtubule dysregulation in inflammation by directly affecting a protein known to be responsible for microtubule stabilization independently of other pathology. Previous studies have shown that *Tppp − / − *cells have WT levels of CFTR activity^21^. The hypothesis of this study is that disruption of microtubule stability through knockout of the Tppp gene will result in a hyperinflammatory state. *Tppp − / − *mice offer the opportunity to study a mouse model of microtubule instability and the effects of microtubule instability on inflammation in the absence of other disease processes.

## Results

### Evaluation of response to airway infection challenge with *P. aeruginosa* in *Tppp* − / − mice

Previous work demonstrated that inflammatory responses in CF mice (mice homozygous for the F508del mutation on CF57Bl/6 background) could me normalized by depleting expression of histone deacetylase 6 (HDAC6)^5^. HDAC6 deacetylates tubulin impacting stability and microtubule-dependent transport^22,23^. However, HDAC6 also has other substrates so it is unclear whether microtubule regulation is responsible for the inflammatory regulation in CF mice. To test the hypothesis that microtubule function is key to inflammatory regulation, we tested in vivo inflammatory responses in a mouse model of microtubule disruption, *Tppp − / − *mice. Mice were inoculated with 25,000 CFU of the clinical isolate of *P*. *aeruginosa* labeled with mCherry (mCH PA M57-15) as described in “Methods”.

#### Weight changes

**Figure 1:**
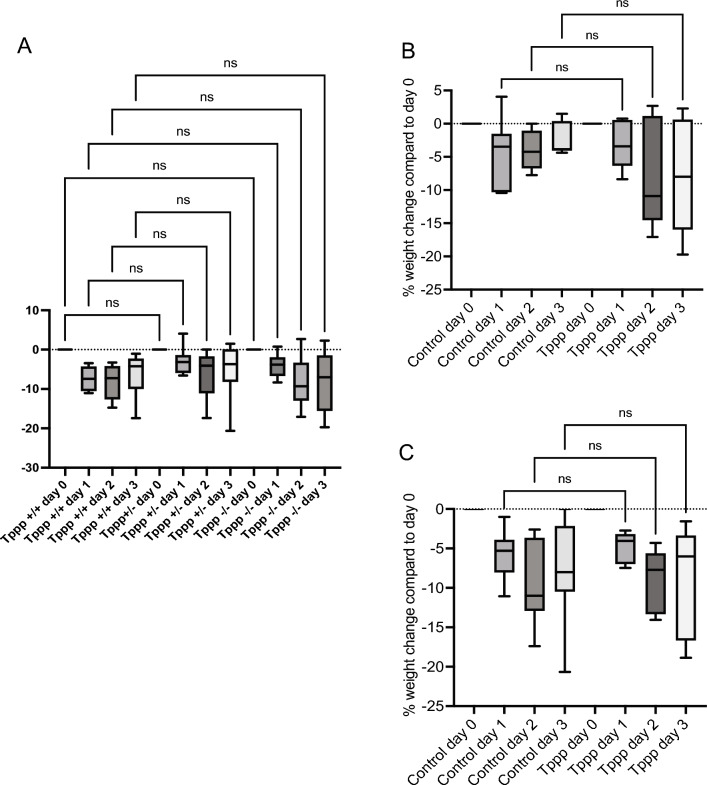
Weight loss on in control (*Tppp* + */* + *; Tppp* + / −) and *Tppp* − / − (Tppp) mice in response to *Pseudomonas aeruginosa* airway infection. Weights were measured at day 0 and days 1, 2 and 3 post-infection for (**A**) *Tppp *+ / + (n = 10), *Tppp* + / − (n = 10) and *Tppp* − / − (n = 10) mice; .Significance determined by ANOVA with Sidak’s multiple comparison test. Significance determined by comparing to Tppp + / + group at each day. (**B**) all female *Tppp* + */* + *; Tppp* + */ − *(control) (n = 13) and Tppp − / − (Tppp) (n = 5) mice; and (**C**) male control (n = 7) and *Tppp* (n = 5) mice. Control mice consist of *Tppp* + */* + and *Tppp* + */ − *mice. Significance determined by ANOVA with Tukey’s multi[le comparison test to determine significance between groups; ns = not significant.

Weight loss is a general measure of the severity of reaction to infection in mice. Control (*Tppp* + / + and *Tppp* − / −) and *Tppp − / − *mice were exposed to *P. aeruginosa* via trans-tracheal inoculation as described in Methods and weight loss determined over three days. No significant differences are observed between control and *Tppp − / − *mice (Fig. 1A). Responses were further broken down to compare male and female mouse response, and there were still no significant differences between control and *Tppp − / − *groups (Fig. 1B,C). Female mice in both groups, however, did lose more weight than male counterparts. These data demonstrate that depletion of Tppp expression has no gross adverse effects on the health of mice in response to airway bacterial challenge.

#### Bacterial clearance

**Figure 2:**
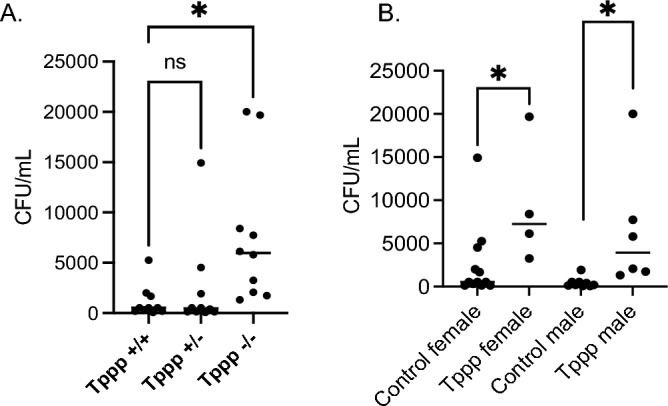
Total colony forming units (CFU) recovered from both bronchoalveolar lavage (BAL) fluid and lung homogenates in *Tppp* + */* + and *Tppp* + */ − *(control)) and *Tppp − / − *(Tppp) mice in response to *Pseudomonas aeruginosa* airway infection. CFU were measured at day 3 post-infection for (**A**) Tppp + / + (n = 10), *Tppp* + */ − *(n = 10) and Tppp − / − (n = 10) mice. Significance determined by ANOVA with Dunnett’s multiple comparison test. Significance determined by comparing to *Tppp* + */* + group. (**B**) all female Control (n = 13) and Tppp (n = 5) mice; and male Control (n = 7) and Tppp (n = 5) mice. Significance determined by unpaired t-test; *p < 0.05.

Previous work demonstrated that HDAC6 depletion in CF mice improved the rates of bacterial clearance compared to CF mice suggesting that correction of the microtubule defect in CF mice had a direct influence on immune response and infection control^5^. To test whether microtubule dysregulation could influence bacterial clearance independently of CFTR function, we examine the levels of recovered CFU 3 days post infection. To address the rate of bacterial clearance in mice, total bacteria (CFU/mL) recovered from both bronchoalveolar lavage (BAL) fluid and lung homogenate at 3 days post infection was assessed. Day 3 was chosen as previous studies have shown that this time point is the peak inflammatory response^5^. In *Tppp − / − *mice, a mean of 7613 + / − 2182 CFU/mL were recovered compared to 1133 + / − 503 CFU/mL for *Tppp* + / + mice and 2333 + / − 1467 CFU/mL for *Tppp* + / − mice (Fig. 2A). Isolating female and male responses (Fig. 2B) show the same reduced clearance in *Tppp − / − *mice regardless of gender. These results demonstrate a reduced rate of bacterial clearance in *Tppp − / − *mice compared to WT mice suggesting a notably reduced ability to handle acute bacterial infection.

#### Inflammatory response to acute *P. aeruginosa* pulmonary infection

**Figure 3:**
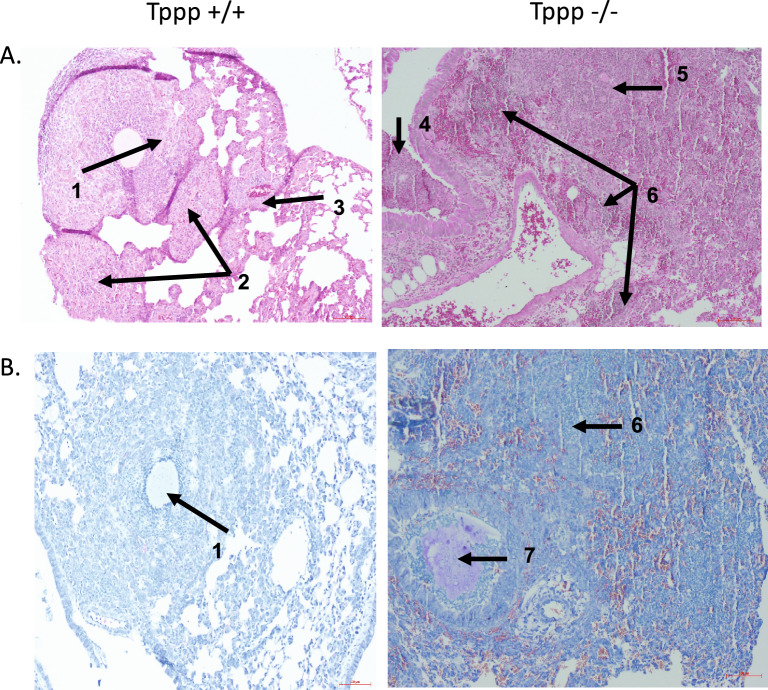
Representative histology images of *Tppp* + */* + and *Tppp − / − *lungs 3-days post-infection with (**A**) hematoxylin and eosin stain and (**B**) Wright-Giemsa stain. 1. Macrophage and neutrophilic inflammatory infiltrate around bacterial embedded agarose bead. 2. Macrophage and neutrophilic infiltration of alveolar spaces and septa. 3. Lymphocytic cuffing of vasculature. 4. Intrabronchial neutrophilic inflammation. 5. Macrophage and neutrophilic infiltration of the alveolar spaces and septa with mild edema. 6. Neutrophilic infiltration of alveolar spaces. 7. Intrabronchial neutrophilic inflammation surrounding bacterial embedded agarose bead.

The hypothesis of the study is that microtubule dysfunction mediated by the lack of Tppp expression would lead to an elevated inflammatory response. To directly test this hypothesis, mice were infected with *P. aeruginosa*-laden agarose beads and evaluated 3-days post-infection as previously reported^5^. Total white blood cell count (WBC) was determined from bronchoalveolar lavage (BAL) fluid from the mice described in Fig. 2. Figure 3 shows representative images of hematoxylin stained (3A) and Wright-Giemsa stained (3B) lung sections at 10X magnification 3-days post *P. aeruginosa*-laden agarose beads in WT and *Tppp − / − *mice. Pathological scoring shows no gross differences in response to infection between groups (Table 1). Samples were scored in a blinded fashion by a pathologist.

**Table 1:** Pathology scoring of airways 3-days post-infection in WT and Tppp − / − mice.

Mouse	Neutrophilic and Macrophage infiltrate Score (1–4)	Peribronchiolar and perivascular lymphocytic cuffing (0–3)	Alveolar septal inflammation (0–3)	Suppurative bronchitis/bronchiolitis (right lung) (0–3)
*Tppp* + / +	4.0 + 0.0	2.7 + 0.2	2.3 + 0.2	1.8 + 0.2
*Tppp* − / −	4.0 + 0.0	2.0 + 0.3	2.2 + 0.2	1.8 + 0.2

Neutrophil scoring scale: Neutrophilic infiltration—1, Absent to rare solitary neutrophils; 2, detectable extravasated neutrophils seen as small loose cellular aggregates in one to a few airways and/or alveoli; 3, detectable extravasated neutrophils seen as loose to compact cellular aggregates in multiple to coalescing airway and/or alveoli with some effacement of lung architecture; 4, detectable extravasated neutrophils seen as compact cellular aggregates effacing most adjacent lung architecture^24^.

**Figure 4:**
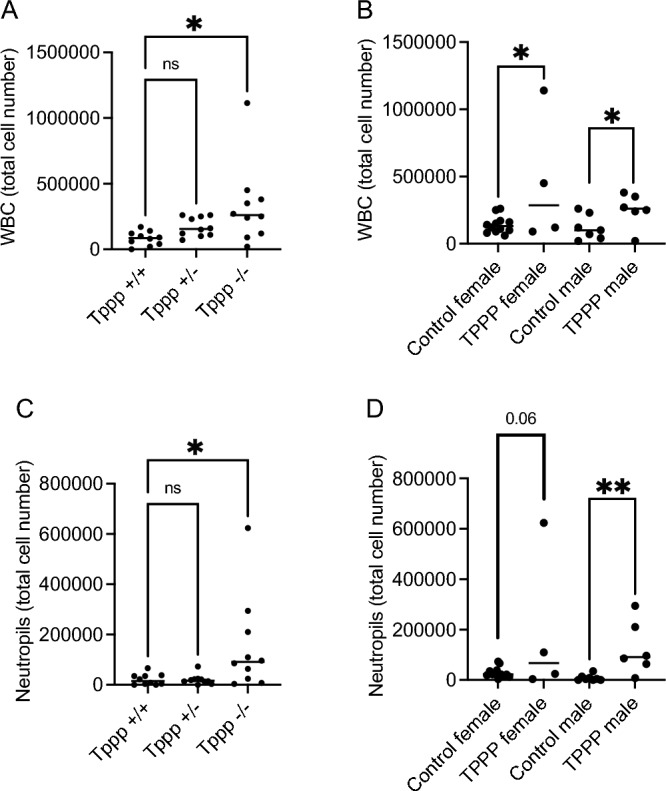
Total white blood cells (WBC) and neutrophils recovered from bronchoalveolar lavage (BAL) fluid homogenates in *Tppp* + / + and *Tppp* + / − (control) and *Tppp − / − *(Tppp) mice in response to *Pseudomonas aeruginosa* airway infection. Total WBC were measured at day 3 post-infection for (**A**) *Tppp *+ / + (n = 10), *Tppp* + / − (n = 10) and Tppp (n = 10) mice; (**B**) all female control(n = 13) and Tppp (n = 5) mice and male control (n = 7) and Tppp (n = 5) mice. Control mice consist of *Tppp* + */* + and *Tppp* + */ − *mice. Significance determined by ANOVA with Dunnett’s multiple comparison test compared to the Tppp + / + group for A and by unpaired t-test between control and TPPP groups for both male and female mice for B. Total neutrophils were measured at day 3 post-infection for (**C**) *Tppp* + / + (n = 10), *Tppp* + / − (n = 10) and Tppp (n = 10) mice; (**D**) all female control(n = 13) and Tppp (n = 5) mice and male control (n = 7) and Tppp (n = 5) mice. Control mice consist of *Tppp* + */* + and *Tppp* + */ − *mice. Significance determined by ANOVA with Dunnett’s multiple comparison test for C and by unpaired t-test between control and TPPP groups for both male and female mice for (**D**); *p < 0.05, **p = 0.01.

Though no gross pathological scoring difference between groups was observed at this acute infection time point, inflammatory responses were quantified by examining bronchoalveolar lavage (BAL) samples. Total white blood cell (WBC) content was found to be significantly greater in *Tppp − / − *compared to control mice (Fig. 4A). Breaking down responses in male and female mice demonstrate a significant increase in total WBC in female *Tppp − / − *mice, but not in male mice though the trend was similar (Fig. 4B).

CF airway inflammatory responses are characterized in part by excessive neutrophil recruitment^25^. It was hypothesized that *Tppp − / − *mice would also exhibit a neutrophil dominant inflammatory response as well. Neutrophil counts from *Tppp − / − *mice was found to be significantly greater than in *Tppp* + / + mice (Fig. 4C). *Tppp* + / − did not differ from *Tppp* + / + mice. Male *Tppp − / − *mice had a significant increase in neutrophils compared to controls while female *Tppp − / − *mice did not, though the trends show a similar increase (Fig. 4D).

**Figure 5:**
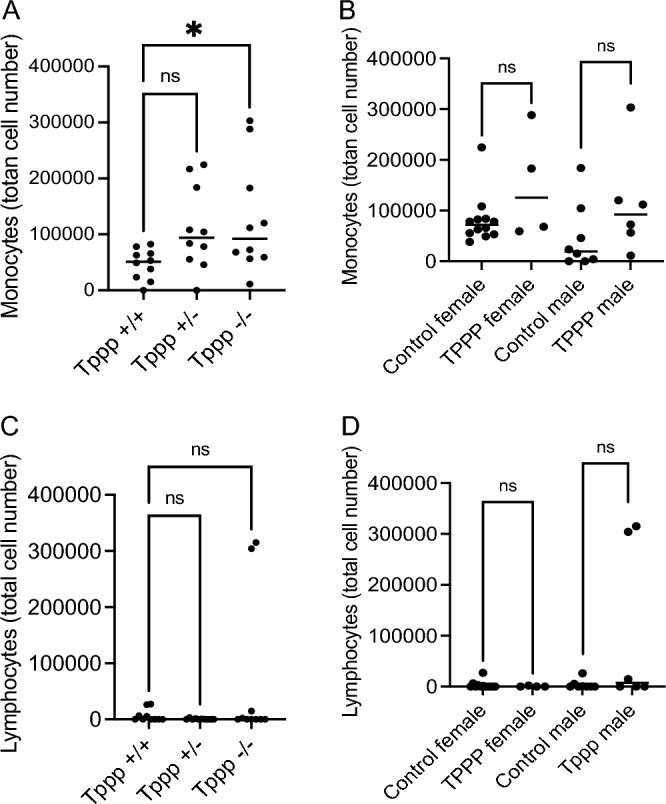
Total monocyte and lymphocyte recovered from bronchoalveolar lavage (BAL) fluid homogenates in *Tppp* + / + and *Tppp* + / − (control)) and *Tppp − / − *(Tppp) mice in response to *Pseudomonas aeruginosa* airway infection. Total monocytes were measured at day 3 post-infection for (**A**) *Tppp* + / + (n = 10), Tppp + / − (n = 10) and Tppp (n = 10) mice; (**B**) all female control (n = 13) and Tppp (n = 5) mice and male control (n = 7) and Tppp (n = 5) mice. Control mice consist of *Tppp* + */* + and *Tppp* + */ − *mice. Significance determined by ANOVA with Dunnett’s multiple comparison test compared to the *Tppp* + / + group for A and by unpaired t-test between control and TPPP groups for both male and female mice for (**B**). Total lymphocytes were measured at day 3 post-infection for (**C**) *Tppp* + / + (n = 10), Tppp + / − (n = 10) and Tppp (n = 10) mice; (**D**) all female control (n = 13) and Tppp (n = 5) mice and male control (n = 7) and Tppp (n = 5) mice. Control mice consist of *Tppp* + */* + and *Tppp* + */ − *mice. Significance determined by ANOVA with Dunnett’s multiple comparison test for C and by unpaired t-test between control and TPPP groups for both male and female mice for (**D**); *p < 0.05, **p = 0.01.

BAL levels of monocytes and lymphocytes were also examined. Monocytes do show a significant increase in *Tppp − / − *BAL fluid after infection compared to Tppp + / + mice consistent with a more aggressive inflammatory response (Fig. 5A). Monocytes in *Tppp* + / − mice did trend higher, but did not reach statistical significance compared to *Tppp* + / + values. Isolating male and female responses show the same trends, but statistical significance is not achieved (Fig. 5B). Lymphocytes levels were generally low in both groups with the exception of two *Tppp − / − *samples leading to a statistically significant change, but with great variability (Fig. 5C). Only male mice showed the elevated lymphocyte counts, but no statistical significance was found (Fig. 5D).

Together these data demonstrate a more robust inflammatory response that is neutrophil dominant in *Tppp − / − *mice compared to controls. Male and female responses show that patterns are the same in each group and that overall significance in the combined analyses is not due to a preferred response in one gender.

#### Cytokine production

**Figure 6:**
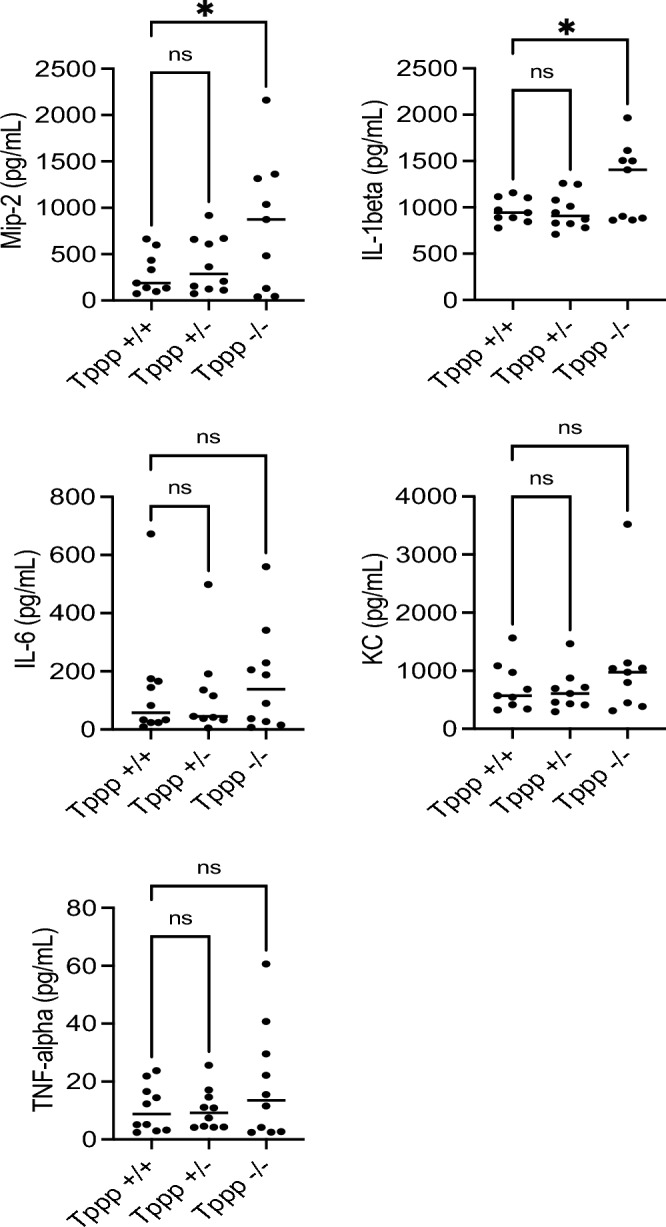
Cytokine levels recovered from bronchoalveolar lavage (BAL) fluid in *Tppp* + */* + (n = 10), *Tppp* + */ − *(n = 10) and *Tppp − / − *(Tppp) mice in response to *Pseudomonas aeruginosa* airway infection. Cytokines were measured at day 3 post-infection for all WT (n = 20) and Tppp (n = 10) mice used. Significance determined by ANOVA with Dunnett’s multiple comparison test compared to the *Tppp* + / + group; *p < 0.05; *ns* not significant.

Cytokine production in response to *P. aeruginosa* infection was examined to further characterize the inflammatory response in *Tppp − / − *mice. Pro-inflammatory cytokines Mip-2, IL-1β, KC, and TNF-α were measured. The production of IL-6 that can have both pro- and anti-inflammatory functions was also measured. Mip-2 and IL1-β cytokines were significantly elevated in lung homogenates from *Tppp − / − *mice compared to Tppp + / + mice. Tppp + / − mice were not different from Tppp + / + mice in any measure (Fig. 6). IL-6 and TNF-a trend higher but did not reach significance. These data support the hypothesis that inflammatory responses are elevated in *Tppp − / − *mice and are consistent with inflammatory cell recruitment data in Figs. 4 and 5. It is unexpected that KC levels are not significantly elevated in *Tppp* − / − mice since it is typically produced in conjunction with Mip-2 in murine epithelium and key to the recruitment of neutrophils as is Mip-2. Cytokine production in male and female mice showed the same patterns but did not reach statistical significance in any group (not shown).

## Discussion

Microtubule disruption is implicated in various disease states as a mechanism of comorbid inflammation in genetic, cardiovascular, and neurologic diseases^2–4^. Previous work of ours demonstrates distinct changes to microtubule regulation in CF cells consisting of reduced tubulin acetylation and slower rates of microtubule reformation^8,9^. We have also demonstrated that knocking down expression of *Tppp* in an immortalized epithelial cell line reproduced these microtubule phenotoypes, consistent with the function of Tppp as an enhancer of microtubule elongation and as an HDAC6 inhibitor^12,20^. Inhibition of HDAC6 has been shown to have significant benefit in addressing multiple CF phenotypes^5,21,26^. Tppp has also been identified as a candidate modifier gene associating with CF airway disease severity in GWAS studies^27^. These GWAS studies directly implicate the Tppp/HDAC6 pathway in CF pathogenesis. The *Tppp − / − *mouse offers a model system to understand the consequences of a CFTR-independent perturbation in this pathway and to test directly the impact of a potential modifier of CF airway disease severity on airway inflammation in response to infection.

The hypothesis of this study was that depletion of Tppp expression in a mouse model would lead to elevated inflammation in response to airway bacterial challenge. This hypothesis stems from previous observations that Hdac6 depletion from CF mice normalized responses to this challenge suggesting a microtubule-related mechanism^5^. We have also recently demonstrated that *Tppp − / − *mice effectively replicate another phenotype of CF mice, circadian timing dysregulation^21,28^. To determine if the Tppp/HDAC6 pathway could be responsible for CF-related airway inflammation, we examined *Tppp − / − *mice. We have previously shown that *Tppp − / − *MNE cells have WT levels of CFTR function so we can conclude that any CF-like phenotypes in these mice are not due to a secondary impact on CFTR activity^21^.

Consistent with the hypothesis, the *Tppp − / − *mice show hypercellular neutrophilic predominant BAL fluids following inoculation with *P. aeruginosa*. Bacterial clearance is impaired in the presence of the hyperinflammatory response, and there are increased CFU cultured in *Tppp − / − *BAL fluids and lung homogenates following inoculation compared to control mice. Mip-2 and IL-1b cytokine production is also increased in BAL fluid of *Tppp − / − *mice. These data are consistent with what is seen in CF mice and demonstrate that disruption of the Tppp/HDAC6 pathway can lead to CF-like inflammatory responses in the presence of WT CFTR function.

Though *Tppp* + / + and *Tppp* + / − groups were not statistically different in any comparison, it was noteworthy that Tppp + / − mice did have monocyte levels that trended higher than *Tppp* + / + mice and were similar to *Tppp* − / − mice. This trend towards elevated monocytes in *Tppp* + / − mice may be statistical variability or reflect a more sensitive outcome measure of innate changes in Tppp expression. Little is known about Tppp and inflammation, so it is difficult to speculate as to why monocytes are trending towards elevated in *Tppp* + / − mice. The trend seems similar in male and female mice, so a gender impact seems unlikely. In our previous work showing circadian disruptions in CF (F508del) mice and *Tppp* − / − mice, we did not examine the impact of *Tppp* heterozygosity on circadian regulation^21,28^. It is well known that sleep disruption can increase inflammation, so it is possible that *Tppp* + / − mice have a level of sleep disruption that is sufficient to impact monocyte recruitment^29^.

In conclusion, these studies demonstrate in an in vivo model that depletion of the microtubule regulatory protein Tppp leads to a CF-like airway inflammatory response. Though these studies do not establish directly that CF inflammation is solely due to microtubule alterations, these studies in conjunction with previous work showing microtubule alterations in CF cells and the efficacy of HDAC6 depletion in reversing CF inflammation help support targeting microtubule regulation as an anti-inflammatory therapeutic target. Since Tppp is an HDAC6 inhibitor, it is possible that the impact of Tppp knockout is independent of microtubule regulation and due to other aspects of HDAC6 signaling. We have previously shown that reducing Tppp expression in epithelial cells recapitulates CF-like inflammatory signaling^12^, therefore future studies will focus on the Tppp/HDAC6 pathway in macrophages and neutrophils function in modulating in vivo inflammatory responses.

## Methods

### Mice

Mice were cared for in accordance with Case Western Reserve University Institutional Animal Care and Use Committee guidelines by the CF Animal Core Facility. The Animal Care and Use Committee of Case Western Reserve University approved all animal procedures. The mouse strain used for this research project, C57BL/6N-*Tppp*^*tm1.1(KOMP)Vlcg*^/JMmucd, RRID:MMRRC_050196-UCD, was obtained from the Mutant Mouse Resource and Research Center (MMRRC) at University of California at Davis, an NIH-funded strain repository, and was donated to the MMRRC by The KOMP Repository, University of California, Davis; Originating from Stephen Murray, The Jackson Laboratory. The mutation is a deletion described at http://velocigene.com/komp/detail/12652.

#### Preparation of *P. aeruginosa*-laden agarose beads

*P. aeruginosa* labeled with mCherry (mCH PA M57-15) was streaked on a 0.3 mg/ml gentamycin/LB agar plate and incubated at 37 °C overnight. The clinical isolate was obtained from the CF Center Biorepository at Rainbow Babies and Children’s Hospital. Five colonies of mCH PA M57-15 were collected and added to 25 ml of LB broth and incubated at 37 °C, 200 rpm, for 18–20 h. After 20 h, the optical density at 600 nm of the mCH PA M57-15 was measured to obtain an absorbance of ~ 0.3 for a tenfold dilution. Bacteria were stored on ice until ready to use.

Two 600-mL beakers containing 250 mL sterile mineral oil and stir bars and a flask of sterile 2% agarose in PBS were warmed in a 50 °C water bath for 30 min. The 2 beakers of mineral oil were placed in ~ 6 × 10 inch plastic container and then placed on magnetic stir plates and set at a medium–high stir to form a vortex. 23 mL of warm, dissolved agarose/PBS was pipetted into one of the beaker of mineral oil to form sterile beads. 5 mL of mCH PAM57-15 was pipetted into the remaining agarose/PBS and mixed and then 23 mL of the mCH PAM57-15/agarose/PBS mixture was pipetted into the other beaker of mineral oil. After 6 min of stirring, 20 g of ice was added to the plastic container every minute for 10 min while monitoring the stirring to ensure a constant vortex. After 10 min, the bead/oil mixture was poured into 50 mL conical tubes containing 15 mL of pre-warmed 0.5% SDC/PBS and spun at 4 °C, 3000 g, for 15 min to separate the beads on the bottom from the top layer of oil. The oil was removed and the top half layer of beads were pipetted into 50 ml conical tubes containing 20 mL of pre-warmed 0.25% SDC/PBS and spun under same conditions. After the second wash, any oil/PBS was removed to ~ 15 mL and the beads were pipetted into 50 ml conical tubes containing 20 mL of PBS and washed 4 ×. After the final wash, the PBS was removed to a 3:4 volume of beads to PBS. A 100 ul aliquot of the bead mixture was used to image and measure the size of beads, which should be ~ 20–600 µm. The beads were viewed under the red channel to ensure sterile beads are sterile and bacterial-laden beads have mCH PA M57-15. A 1:50 dilution of the beads was homogenized on highest speed for 1 min and serial diluted tenfold 10 times. The dilutions were plated on a 0.3 mg/mL gentamycin/LB agar plate and incubated at 37 °C overnight. The following day, the CFU/mL were calculated the bead/PBS mixture was diluted to a concentration of 25,000 CFU/50 µL.

#### Inoculation of mouse airway with agarose beads

All animal studies were approved by the CWRU IACUC committee, and all experiments were performed in accordance with relevant guidelines and regulations. The mice receiving sterile bead treatment were used first to avoid cross contamination. The mouse was anesthetized with isoflurane via inhalation with a nosecone. Ophthalmic ointment was applied the eyes and the thorax was sterilized with isopropanol. A ~ 1 cm midline median incision was made using surgical scissors in the tracheal region. The tracheal muscles were gently teased apart to expose the trachea and a ~ 1 mm transverse incision was made between the tracheal cartilage. A 1 mL syringe fitted with a 22G × 1″ flexible catheter was used to mix and draw up the bead/PBS mixture leaving no dead space in catheter. The catheter/syringe was immediately inserted into the tracheal incision and 50 mL of the bead/PBS mixture was injected into the lungs of the mouse to administer 25,000 CFU per mouse. The mouse was placed into a clean, sterile cage on top of a warming pad and monitored until out of anesthesia. The weight and condition of inoculated mice were monitored until the day of harvest.

#### Harvest

Sterile bead inoculated mice were harvest first to avoid cross contamination. The mouse was euthanized by CO2 inhalation, placed in the supine position, and disinfected with 70% ethanol. A midline, median incision was made to expose the thoracic cage and the diaphragm cut to expose the lungs. A 22G × 1″ flexible catheter was inserted between the tracheal cartilage to just before the bronchi. Surgical thread was tied around trachea and catheter. BAL fluid was collected using a syringe filled with 1 ml PBS inserted into the catheter and injected into and withdrawn from lungs. Ten microliters of BAL fluid was used to determine total white blood cell count, another 15 µL was plated on 0.3 mg/mL gentamycin/LB agar for CFU/µL count, and 200 µL of BAL fluid was used to determine cell differentials using Giemsa/Miller staining. The BAL fluid was then spun at 4 °C, 500 g, 10 min. The supernatant was used for cytokine analysis using Luminex. The lungs were removed and rinsed with PBS and placed in 2 mL tubes containing zirconium homogenization beads and homogenized and 15 µL plated on 0.3 mg/mL gentamycin/LB agar for CFU/µL count. The remaining lung homogenate was spun at 4 °C, 5000 g, 10 min and the supernatant removed. The remaining supernatant was stored at − 80 °C. For staining of lung tissue, 10 μm sections were fixed in formalin, embedded in paraffin, deparaffinized in xylene and ethanol, and stained with hematoxylin and eosin by the histology core facility at CWRU.

### White blood cell differentiation and counting

Cell counts were conducted manually by a board-certified pathologist blinded to experimental group through the CF Histology Core facility at CWRU. Cells are counted manually in a Neubauer chamber and morphology determined visually. If necessary histological staining is performed to differentiate between neutrophils, lymphocytes, and leukocytes.

### Statistical analysis

Statistical analyses were performed with GraphPad-Prism, Version 10.0.0. Analysis of results with Tppp + / + , Tppp + / − , and Tppp − / − groups were done by ordinary one-way ANOVA with Dunnett’s multiple comparison test to compare Tppp + / − and Tppp − / − groups to the Tppp + / + group; p ≤ 0.05 is * and p ≤ 0.01 is **. Statistical analysis of male and female groups were done with unpaired t-test comparing control group (Tppp + / + and Tppp + / − groups) to Tppp − / − groups; p ≤ 0.05 is * and p ≤ 0.01 is **.

### ARRIVE guidelines

This study has been conducted and data reported in accordance with ARRIVE guidelines.

## Data Availability

The datasets generated during and/or analyzed during the current study are available from the corresponding author on reasonable request.
